# Supplementary material for: Factors impacting employee turnover intentions among professionals in Sri Lankan startups
Source: PLoS One. 2023 Feb 10;18(2):e0281729. doi: 10.1371/journal.pone.0281729 (PMC9916568; doi:10.1371/journal.pone.0281729)
Supplement: S1 Appendix — (DOCX) [file pone.0281729.s001.docx]

# S1 Appendix. Questioner

**Employees thoughts who work for Sri Lankan Startups**

Dear sir/madam,

If you are working or have worked for any Sri Lankan startup, we would like to have your valuable response and it will guide us to the success of this academic research.

This information is a part of my academic research which attempts to study the factors to consider about employees in Sri Lankan startups. Your responses will be strictly confidential. To complete this questionnaire, it will take a maximum of 10 minutes only. Your participation is important to the success of this research and your contribution is highly appreciated. Your professional assistance will help me to complete this academic research and to add something new to our society.

Thank you!

Y. L. Kanchana

Postgraduate student

Master of Business Administration

Institute of Information Technology

1. **Part 01 - General Information**

kindly put a tick to the appropriate category

- 1. **Gender identity**

| Male |  | Female |  |
| --- | --- | --- | --- |

- 1. **Age group (in years)**

| below 20 |  |
| --- | --- |
| 20 - 30 |  |
| 31 - 40 |  |
| 41 – 50 |  |
| above 50 |  |

- 1. **Educational/ Professional qualifications**

(Please mark the higher Educational/ Professional qualification received)

| No schooling |  |
| --- | --- |
| Passed grade 9 |  |
| Passed G.C.E. O/L or G.C.E A/L or equivalent |  |
| Passed certificate or diploma level |  |
| Passed Degree |  |
| Passed post graduate |  |
| PHD |  |

- 1. **What is your work status**

| Full time |  | Part time |  |
| --- | --- | --- | --- |

|  |
| --- |

- 1. **The district you are living in**

(Colombo, Gampaha, Kalutara, Kandy, Matale, Nuwara Eliya, Galle, Matara, Hambantota, Jaffna, Kilinochchi, Mannar, Vavuniya, Mullaitivu, Batticaloa, Ampara, Trincomalee, Kurunegala, Puttalam, Anuradhapura, Polonnaruwa, Badulla, Moneragala, Ratnapura, Kegalle)

1. **Job Satisfaction**

|  | **Rarely** | **Sometimes** | **Every once in a while** | **Often** | **Always** |
| --- | --- | --- | --- | --- | --- |
| - 1. I am given the resources and equipment to do my job. |  |  |  |  |  |
| - 1. I am happy about what I physically receive (materials and monitory rewards) from the workplace. |  |  |  |  |  |
| - 1. I would strongly endorse this company to my friends and family to join. |  |  |  |  |  |
| - 1. I recommend my workplace as a physically safe place to work. |  |  |  |  |  |
| - 1. I feel I receive a fair share of the profits made by this organization. |  |  |  |  |  |
| - 1. People here are paid fairly for the work they do. |  |  |  |  |  |
| - 1. My work has a special meaning: this is not "just a job". |  |  |  |  |  |
| - 1. Company facilities contribute to a good working environment. |  |  |  |  |  |
| - 1. I am happy about the life insurance that company has offered. |  |  |  |  |  |

1. **Work-life balance**

|  | **Rarely** | **Sometimes** | **Every once in a while** | **Often** | **Always** |
| --- | --- | --- | --- | --- | --- |
| - 1. People are encouraged to balance their work life and their personal life. |  |  |  |  |  |
| - 1. I can request a leave whenever I need, and management fairly considers my request. |  |  |  |  |  |
| - 1. I am okay with working hours. |  |  |  |  |  |
| - 1. I got to know about many people through this job. |  |  |  |  |  |
| - 1. I can take time off from work when I think it is necessary. |  |  |  |  |  |

1. **Happiness**

|  | **Rarely** | **Sometimes** | **Every once in a while** | **Often** | **Always** |
| --- | --- | --- | --- | --- | --- |
| - 1. I feel good about the ways we contribute to the community. |  |  |  |  |  |
| - 1. My organization celebrates special occasions. |  |  |  |  |  |
| - 1. I am happy about this organization's culture. |  |  |  |  |  |
| - 1. I am proud to tell others I work here. |  |  |  |  |  |
| - 1. When I look at what we accomplish, I feel sense of pride. |  |  |  |  |  |

1. **Management support**

|  | **Rarely** | **Sometimes** | **Every once in a while** | **Often** | **Always** |
| --- | --- | --- | --- | --- | --- |
| - 1. Management recognizes my honest mistakes as part of doing business. |  |  |  |  |  |
| - 1. I can ask management any reasonable question and get a straight answer. |  |  |  |  |  |
| - 1. Management is easy to talk with. |  |  |  |  |  |
| - 1. Management involves people in decisions that affect their jobs or work environment. |  |  |  |  |  |
| - 1. Management of the organization shows appreciation for good work and extra effort. |  |  |  |  |  |

1. **Career Management**

|  | **Rarely** | **Sometimes** | **Every once in a while** | **Often** | **Always** |
| --- | --- | --- | --- | --- | --- |
| - 1. I am offered training and development to further myself professionally. |  |  |  |  |  |
| - 1. Here, everyone has an opportunity to get special recognition. |  |  |  |  |  |
| - 1. You receive opportunities to learn something new from the organization. |  |  |  |  |  |
| - 1. I am satisfied with my career progress. |  |  |  |  |  |

1. **Innovative Work Behavior (IWB)**

|  | **Rarely** | **Sometimes** | **Every once in a while** | **Often** | **Always** |
| --- | --- | --- | --- | --- | --- |
| - 1. I feel I make a difference here. |  |  |  |  |  |
| - 1. Your company celebrate new creations and ways to do things in the workplace. |  |  |  |  |  |
| - 1. I had meaningful opportunities to develop new and better ways of doing things at work. |  |  |  |  |  |
| - 1. When I try something new at work and others help and motivate me. |  |  |  |  |  |

1. **Leader Member Exchange (LMX)**

|  | **Rarely** | **Sometimes** | **Every once in a while** | **Often** | **Always** |
| --- | --- | --- | --- | --- | --- |
| - 1. My supervisors support me whenever I need them. |  |  |  |  |  |
| - 1. People in this workplace are willing to give extra to get the job done. |  |  |  |  |  |
| - 1. My supervisors have a positive attitude. |  |  |  |  |  |
| - 1. I am comfortable talking with my supervisor about challenges I face at work. |  |  |  |  |  |

1. **Co-worker Support**

|  | **Rarely** | **Sometimes** | **Every once in a while** | **Often** | **Always** |
| --- | --- | --- | --- | --- | --- |
| - 1. My co-workers support me whenever I need them. |  |  |  |  |  |
| - 1. People care about each other here. |  |  |  |  |  |
| - 1. When there is a teamwork, my co-workers are staying with me until the end of our assigned task. |  |  |  |  |  |
| - 1. My co-workers are treated fairy to me regardless of my age. |  |  |  |  |  |

1. **Employee Turnover**

|  | **Rarely** | **Sometimes** | **Every once in a while** | **Often** | **Always** |
| --- | --- | --- | --- | --- | --- |
| - 1. How often have your seriously considered quitting the job in this workplace? |  |  |  |  |  |
| - 1. If you receive a job opportunity from another company, how often you would consider the opportunity seriously. |  |  |  |  |  |
| - 1. Almost nothing in my life would be disrupted if I decided to leave my organization now. |  |  |  |  |  |
| - 1. My team members leave the organization regularly. |  |  |  |  |  |

Thank you for taking time to complete this questionnaire!
