# Supplementary material for: Factors impacting employee turnover intentions among professionals in Sri Lankan startups
Source: PLoS One. 2023 Feb 10;18(2):e0281729. doi: 10.1371/journal.pone.0281729 (PMC9916568; doi:10.1371/journal.pone.0281729)
Supplement: S3 Appendix — (DOCX) [file pone.0281729.s003.docx]

# S3 Appendix. Initial Ordered Probit Regression Results

| **Variable** |  | | **Marginal effects (in percentages)** | | |
| --- | --- | --- | --- | --- | --- |
|  | **Estimate** | **Robust SE** | **Low ET (*Y=1*)** | **Moderate ET (*Y=2*)** | **High ET (*Y=3*)** |
| lnJS | -1.8769** | 0.7713 | 0.6357** | -0.0493 | -0.5864** |
| lnWLB | 0.7472 | 0. 6461 | -0.2531 | 0.0196 | 0.2335 |
| lnH | 0.7130 | 0.6145 | -0.2415 | 0.0187 | 0.2228 |
| lnMS | -0.8384 | 0.6756 | 0.2840 | -0.0220 | -0.2620 |
| lnCM | -0.8406 | 0.5244 | 0.2847 | -0.0221 | -0.2626 |
| lnIWB | -0.8313 | 0.5370 | 0.2816 | -0.0218 | -0.2597 |
| lnLMX | 1.5768*** | 0.5555 | -0.5341*** | 0.0414 | 0.4927*** |
| lnCWS | -1.2083*** | 0.4252 | 0.4093*** | -0.0318 | -0.3775*** |
| *Socio-demographic characteristics* | | | | | |
| G_Male | 0.4720** | 0.1859 | -0.1661** | 0.0283 | 0.1378*** |
| A_20_30 | 1.1319 | 0.9004 | -0.4251 | 0.1855 | 0.2397** |
| A_31_40 | 1.8656** | 0. 9401 | -0.3213*** | -0.3255* | 0.6469*** |
| A_41_50 | 0.3499 | 1.1192 | -0.1061 | -0.0146 | 0.1206 |
| Edu_Certificate | 0.4800 | 0.4969 | -0.1477 | -0.0152 | 0.1629 |
| Edu_degree | 0.4989 | 0.4916 | -0.1716 | 0.0210 | 0.1506 |
| Edu_postgraduate | 0.6688 | 0.5287 | -0.1939 | -0.0404 | 0.2343 |
| WS_Fulltime | -0.0921 | 0.3727 | 0.0305 | -0.0009 | -0.0295 |
| **Ancillary parameters** | | | **Marginal effects after ordered probit** | | |
| γ̂_1_ | -1.9546 | 1.0481 | 0.2836 | 0.4741 | 0.2422 |
| γ̂2 | -0.6834 | 1.0429 |  |  |  |
| Pseudo R^2^ | 0.7260 | |  |  |  |
| Log likelihood | -194.9551 | |  |  |  |
| Number of observations | 230 | |  |  |  |

Note: *** significant at the 1% level, ** significant at the 5% level and * significant at the 10% level.
