# Supplementary material for: Factors impacting employee turnover intentions among professionals in Sri Lankan startups
Source: PLoS One. 2023 Feb 10;18(2):e0281729. doi: 10.1371/journal.pone.0281729 (PMC9916568; doi:10.1371/journal.pone.0281729)
Supplement: S4 Appendix — (DOCX) [file pone.0281729.s004.docx]

# S4 Appendix. Literature Summary

# Studies on the relationship between job satisfaction and employee turnover

(Table continues the next page)

| **Author(s)** | **Objective(s)** | **Country, type of data and period** | **Methodology** | **Conclusion** |
| --- | --- | --- | --- | --- |
| Kim M, Knutson BJ, Choi L.  2015 | To examine the relational  differences of employee voice, employee delight, satisfaction, loyalty, and turnover intent between Gen Y employees and older employees | Midwest United States,  Gen Y employees and older employees in two hotels, independently owned and operated in the Midwest  April to May 2012 | Structural comparison | Gen Y employees showed lower values of voice, delight, satisfaction, and loyalty than did their older counterparts, while their turnover intent was greater. |
| Da Camara N, Dulewicz V, Higgs M.  2015 | To investigate the relationship between perceptions of organizational emotional intelligence (OEI) and turnover intentions amongst employees. | United Kingdom,  employees in a UK-based charity organization | Partial least squares structural equation modelling (PLS-SEM) | Most of the mediation occurs through job satisfaction with a reduced mediation effect for affective commitment. |
| Lu L, Lu ACC, Gursoy D, Neale NR.  2015 | To examine differences in engagement and its  outcomes between supervisors and line-level employees within the hospitality literature. | North America,  Line-level employees in North American branded hotel management company | One-way ANCOVA | Age has positive impact on employee turnover. Employee positions significantly moderate the relationship between absorption and job satisfaction, and the relationship between dedication and turnover intentions. |
| Oosthuizen RM, Coetzee M, Munro Z.  2016 | To explore the association between employees’ experiences of work-life balance, job satisfaction and their turnover intention | South Africa,  79 permanently employed salaried employees in a South African IT company | Regression analysis | Experiences of negative work-home interaction and positive  work-home interaction significantly predicted job Satisfaction and turnover intention. Job satisfaction also significantly predicted turnover intention. However, no interaction effect was observed between overall work-life balance and job satisfaction in predicting turnover intention. |
| Farooq H, Janjua UI, Madni TM, Waheed A, Zareei M, Alanazi F.  2022 | To identify and empirically evaluate the factors of turnover intention in Pakistan’s software and IT industries | Pakistan,  50 professionals from Pakistan’s IT and software industry | Regression analysis | Recruitment & section, team & management support, performance & career management, salary & compensation, employee commitment, job security, recognition, organizational demographics, and personal demographics with mediating the role of job satisfaction have a significant impact on IT professionals’ turnover intention except for age and gender |
| Zeffane R, Bani Melhem SJ.  2017 | To examine and compare the differential impacts of job satisfaction, trust, and perceived organizational performance on turnover intention in public and private sector organizations. | United Arab Emirates,  311 employees from the service sector | Regression analysis | Public sector employees’ turnover intention (TI) is most significantly affected by their perceptions of the performance of their organization, with JS, work experience and education also having significant effects. In contrast, private sector employees’ TI was most  (Table continues the next page)  significantly affected by JS and feelings of trust. |
| Kaur R, Randhawa G  2020 | To investigate the role of employee engagement and work–life balance in perceived supervisor support and turnover intentions relationship | India,  375 teachers at private schools of Punjab, India | Parallel multiple mediated regression analysis | supervisor support on turnover intentions is indirect rather than direct |
| Thomas A. Wright DGB  2007 | To examine the relationships among psychological well-being, job satisfaction, and employee job performance with employee turnover | United States,  2 years of field study  112 managers employed at a large organization | Logistic regression analysis | Well-being was found to moderate the relation between job satisfaction and job separation. Job satisfaction was most strongly (and negatively) related to turnover when well-being was low. |
| Nae EY, Choi BK.  2021 | To explain the important yet relatively  neglected link between career satisfaction and turnover intention | South Korea,  192 employees | Multiple regression analysis | The indirect relationship between career satisfaction and turnover intention through subjective wellbeing was significant only when employees had high-secure attachment and low-counter dependent and -overdependent attachment styles. |

# Studies on the relationship between work-life balance and employee turnover

(Table continues the next page)

| **Author(s)** | **Objective(s)** | **Country, type of data and period** | **Methodology** | **Conclusion** |
| --- | --- | --- | --- | --- |
| Deery M, Jago L.  2015 | To examine the themes of talent management, work-life balance (WLB) and retention strategies in the hospitality industry | Multiple countries,  Literatures from year 2009 to 2013 | Concept paper | WLB appears to have become one of the key variables when addressing issues of employee management and retention |
| Oosthuizen RM, Coetzee M, Munro Z.  2016 | To explore the association between employees’ experiences of work-life balance, job satisfaction and their turnover intention | South Africa,  79 permanent employees in an IT company | Regression analysis | Experiences of negative work-home interaction and positive work-home interaction significantly predicted job satisfaction and turnover intention. Job satisfaction also significantly predicted turnover intention. |
| Dechawatanapaisal D.  2017 | To investigate the mediating effect of organizational embeddedness in the relationship between quality of work life (QWL) and turnover under a foundation of conservation of resources theory | Thailand,  422 healthcare professionals | Structural equation modelling | Organizational embeddedness has a negative impact on employees’ intention to leave, and on actual turnover. Career opportunities, work life balance, and job characteristics are positive and significant predictors of organizational embeddedness. |
| Kaur R, Randhawa G.  2020 | To investigate the role of employee engagement and work–life balance in perceived supervisor support and turnover intentions relationship | India,  375 teachers at private schools in Panjab | Multiple mediated regression | Supervisor support on turnover intentions is indirect. Employee engagement and two dimensions of work–life balance act as mediators in the perceived supervisor support and turnover intentions relationship |
| Limited EP.  2021 | Explore concerns about unsatisfactory work-life balance is negatively affecting turnover in firms. | Multiple countries,  Literature 2021 | Review of literature | Work-life balance is negatively affecting turnover in many firms. Family-friendly HRM practices that address employee needs can help businesses reverse this trend and achieve desired outcomes from their talent management and employer branding initiatives |
| Cain L, Busser J, Kang HJ  2018 | To understand the relationships among calling, employee engagement, work-life balance and life satisfaction for executive chefs based on role theory and spillover theory. | North America,  members of the American Culinary Federation | Structural equation modelling | All relationships in the model were significantly positive except for calling to life satisfaction. Work-life balance was a significant mediator between calling and life satisfaction, employee engagement and life satisfaction |

# Studies on the relationship between happiness and employee turnover

| **Author(s)** | **Objective(s)** | **Country, type of data and period** | **Methodology** | **Conclusion** |
| --- | --- | --- | --- | --- |
| Wright T, Cropanzano R.  2007 | Revisit happy/productive worker thesis | Multiple countries,  Literatures from 1918 to 2004 | Systematic review | Suggest the consideration of worker happiness as psychological well-being (PWB). Suggest that the job satisfaction to job performance and job satisfaction to employee retention relationships may be better explained by controlling for the moderating effect of PWB |
| Jalilianhasanpour R, Asadollahi S, Yousem DM.  2021 | How to create a joyful workplace | Not given | Qualitative | To achieve a greater sense of joy at work requires attention paid to the physical and psychological environment and should be strategically addressed to augment happiness |

# Studies on the relationship between management support and employee turnover

(Table continues the next page)

| **Author(s)** | **Objective(s)** | **Country, type of data and period** | **Methodology** | **Conclusion** |
| --- | --- | --- | --- | --- |
| van den Heuvel S, Freese C, Schalk R, van Assen M.  2017 | To examine how the quality of change information influences employees’ attitude toward organizational change and turnover intention | United states,  669 employees in a technology services organization | Structural equation modelling | Engagement and psychological contract fulfilment were positively related to attitude toward change and negatively related to turnover intention |
| Li Q, Mohamed R, Mahomed A, Khan H.  2022 | To examine the impact of perceived organizational support and employee care on turnover intention and to test the mediating effect of work engagement on frontline medical staff | China,  533 medical workers  From January to March 2022 | Partial least square (PLS) path modelling | The informal care of managers as a job resource can meet the psychological needs of frontline medical staff in the current environment |
| Saoula O, Johari H.  2016 | To determine the relationship between perceived organizational support, organizational citizenship behaviour and turnover intention | Malaysia,  employees working in the ICT sector | Partial  least square-structural equation modelling | The use of Organizational Citizenship Behaviour in the relationship between  Perceived Organizational Support and turnover intention helps in the early prediction of TI |
| Wong Y-W, Wong Y-t.  2017 | To explore the relationships of turnover intention, perceived organisational support (POS) and affective commitment in China | China,  Foreign-invested enterprise (FIE) manufacturing company | Structural equation modelling | distributive justice, trust in organisation and job security have negative impacts on turnover intention. Affective commitment mediates the impact of job security on turnover intention. |
| Xiu L, Dauner KN, McIntosh CR.  2019 | To examine the relationship between employees’ perceptions of organizational support for employee health (OSEH) and employees’ turnover intention and job performance | United States,  297 employees at a public university | Regression analysis | employees’ perceptions of OSEH positively related to both turnover intention and job performance and that affective commitment fully mediated the relationships between OSEH perceptions and both dependent variables |

# Studies on the relationship between career management and employee turnover

| **Author(s)** | **Objective(s)** | **Country, type of data and period** | **Methodology** | **Conclusion** |
| --- | --- | --- | --- | --- |
| Digest HRMI  2017 | To review the latest management developments across the globe and pinpoint practical implications from cutting-edge research and case studies | Multiple countries,  Literature of 2017 | Case studies | Employee turnover involves significant costs in recruiting and hiring replacements. Companies should adopt a formal approach to talent retention and incorporate it into HR and wider organizational strategies |
| Saoula O, Johari H.  2016 | To examine the impact of perceived organizational support and employee care on turnover intention and to test the mediating effect of work engagement on frontline medical staff | China,  533 medical workers  From January to March 2022 | Partial least square (PLS) path modelling | The informal care of managers as a job resource can meet the psychological needs of frontline medical staff in the current environment |
| Rawashdeh AM, Tamimi SA.  2019 | To investigate the impact of employee perception of training on organizational commitment, and organizational commitment on turnover intention | Jordan,  302 registered nurses working at Jordanian hospitals | Social science analysis of regression | The perceived benefits of training negatively affected to organizational commitment. There is a strong inverse association between organizational commitment and turnover intention |

# Studies on the relationship between innovative work behaviour and employee turnover

| **Author(s)** | **Objective(s)** | **Country, type of data and period** | **Methodology** | **Conclusion** |
| --- | --- | --- | --- | --- |
| Shih HA, Posthuma RA, Susanto E.  2011 | To investigate the negative impacts of innovative work behaviour (IWB) on conflict with co-workers and turnover intention. to test the moderating effect of perceived distributive fairness on these relationships | Indonesia,  460 employees in production and marketing teams at manufacturing and pharmaceutical companies | Multiple hierarchical regressions | Innovative work behaviour had a positive and significant relationship with conflict with co-workers and turnover intention respectively |
| Saoula O, Fareed M, Hamid RA, Al-Rejal HMEA, Ismail SA.  2019 | To examine the relationship between organisational justice (OJ)’s three dimensions (Distributive justice, procedural justice, and interactional justice), organisational learning culture (OLC) and turnover intention (TI) | Malaysia,  full time employees working in the information and communication technology sector | Structural Equation Modelling -Partial Least Square (SEM-PLS) | Identify more insights of turnover intention |
| Agarwal UA, Datta S, Blake‐Beard S, Bhargava S  2012 | To examine the relationships among leader‐member exchange (LMX), innovative work behaviour (IWB), and intention to quit | India,  979 Indian managerial employees working in six service sector organisations | Structural equation modelling | Work engagement correlates positively with innovative work behaviour and negatively with intention to quit. Work engagement mediates the relationship between LMX and innovative work behaviour, and partially mediates intention to quit |

# Studies on the relationship between leader-member exchange and employee turnover

| **Author(s)** | **Objective(s)** | **Country, type of data and period** | **Methodology** | **Conclusion** |
| --- | --- | --- | --- | --- |
| Tobias M. Huning KJH, Rachel E. Frieder  2020 | To provide a theoretical foundation for the effects of servant leadership and to examine the mediating effects of perceived organizational support, job satisfaction and job embeddedness on employees’ turnover intentions | United States,  115 full-time employees from several organizations in a metropolitan area in the south-eastern United States | Corelation analysis | Servant leadership is negatively related to turnover intentions however servant leadership does not have a direct effect on turnover intentions |
| Gyensare MA, Kumedzro LE, Sanda A, Boso N.  2017 | To examine how employee engagement and affective commitment mediate the relationship between transformational leadership and voluntary turnover intention | Ghana,  336 employees in a large public sector organisation | Hierarchical linear modelling with bootstrapping analysis | Transformational leadership positively influenced engagement, which was then negatively related to employee turnover intention |
| Kaur R, Randhawa G.  2020 | To investigate the role of employee engagement and work–life balance in perceived supervisor support and turnover intentions relationship | India,  375 teachers at private schools in Panjab | Multiple mediated regression | Supervisor support on turnover intentions is indirect. Employee engagement and two dimensions of work–life balance act as mediators in the perceived supervisor support and turnover intentions relationship. |
| Tymon WG, Stumpf SA, Smith RR  2011 | To examine how the support managers provide to employees affects the employees' sense of intrinsic reward, personal commitment, perceived career success, and retention | India,  4,811 employees | Path analytic regression | Managerial support of employees had significant direct and indirect effects on perceived career success and retention one year later |

# Studies on the relationship between co-worker support and employee turnover

| **Author(s)** | **Objective(s)** | **Country, type of data and period** | **Methodology** | **Conclusion** |
| --- | --- | --- | --- | --- |
| Kmieciak R.  2021 | To assess the effect of co-worker support on horizontal knowledge withholding and voluntary turnover intention among IT specialists. Explore the mediating role of affective organizational commitment. | Poland,  118 IT specialists from a Polish software company  In November 2019 | Structural model | Negative effect of co-worker support on voluntary turnover intention is fully mediated by organizational affective commitment. Contrary to expectations, co-worker support is not significantly negatively related to horizontal knowledge withholding. |
| Abugre JB, Acquaah M.  2022 | To evidently examine how employee cynicism mediates the relationship between co-worker relationship and employee turnover intentions in organizations in Ghana | Ghana,  288 employees | Structural equation modelling | Co-worker relationship is negatively associated with employee cynicism |
